# Reply to “Limitations of repurposing ceftazidime for *Stenotrophomonas maltophilia*: a cautionary perspective”

**DOI:** 10.1128/msphere.00404-25

**Published:** 2025-08-04

**Authors:** Matthew C. Phillips, Rosemary She, Brad Spellberg, Brian Luna

**Affiliations:** 1 Division of Infectious Diseases, Department of Medicine, Massachusetts General Hospital 571475, Boston, Massachusetts, USA; 2 Harvard Medical School 1811 , Boston, Massachusetts, USA; 3 Department of Pathology, City of Hope 378541 , Duarte, California, USA; 4 Division of Infectious Diseases, Department of Medicine, Los Angeles General Hospital, Los Angeles, California, USA; 5 Department of Molecular Microbiology and Immunology, Keck School of Medicine at USC, Los Angeles, California, USA; University of Nebraska Medical Center College of Medicine, Omaha, Nebraska, USA

## REPLY

We thank Irulappan and Veeraraghavan for their letter (1) and their interest in our work (2). We fully agree that “standard *in vitro* methods may not always reflect *in vivo* outcomes,” and we believe this subject warrants further research.

The authors caution that “ceftazidime (CAZ) activity under metal-depleted conditions may not hold in zinc-rich inflamed tissues, where metallo-β-lactamases like L1 could be reactivated, negating *in vitro* benefits.” However, previous studies have found that inflammation results in a decreased availability of zinc as a host defense strategy, and the abundance of zinc was not significantly different in the tissues of uninfected mice as compared to mice infected with *Acinetobacter baumannii* (3–5).

The authors also write that “the study clearly demonstrates that not all antibiotics benefited equally from the medium shift” and that the “antibiotic-specific nature of media effects and caution against overreliance on CAZ.” We agree that the modified antibiotic susceptibility testing conditions that we used in this study only demonstrated that CAZ, and not other beta-lactams, was generally more active against *Stenotrophomonas maltophilia* clinical isolates. It was notable that CAZ, and not meropenem, was effective against *S. maltophilia* because it has previously been reported that meropenem retained activity against metallo-β-lactamase expressing *Pseudomonas aeruginosa in vitro* in zinc-limited media and *in vivo* in murine infection models as well (6). Therefore, while we identified CAZ as being effective against *S. maltophilia,* it would still need to be empirically tested if CAZ would be effective against other metallo-β-lactamase-producing pathogens.

Additionally, alternative media conditions are already employed in the clinical microbiology lab in an antibiotic-specific way, such as when calcium is added to media for daptomycin susceptibility testing or iron-depleted media is used for cefiderocol. These alternative conditions to traditional CAMHB are necessary to ensure the *in vivo* relevance of the susceptibility testing. The authors state that “RPMI 1640, though mimicking some *in vivo* conditions, lacks validation for routine use and is not standardized by CLSI or EUCAST.” We agree that it is not standard media for use with bacterial organisms, but we point out that RPMI 1640-based media has been recommended by CLSI and EUCAST in their antifungal susceptibility testing standards. Since not all antibiotic resistance mechanisms are dependent on metal cations, we would not expect the media conditions in our study to work equally well for other antibiotics with other mechanisms of resistance. More importantly, there is a growing body of literature, including this current study, that suggests changes in standardized media may be necessary for specific antibiotic *resistance mechanisms*, such as metallo-β-lactamases, similar to how they are necessary for certain antibiotics.

 In response to the concern “about the supratherapeutic dosing used in animal models,” we note that the metabolism of beta-lactam antibiotics in mice results in a much shorter half-life compared to humans. Additionally, beta-lactams do not kill bacteria in a concentration-dependent manner but rather time-dependent based on %fT > MIC (7). Based on previously published pharmacokinetic studies of CAZ in mice (8), we estimate about 26% fT > 4 mg/L (the MIC of the strain used in the mouse model as determined in the zinc-limited broth) for the 300 mg/kg dosing strategy in our study. Thus, our models use a conservative dosing strategy and do not overdose the mice.

Finally, the authors write that “we urge clinicians to maintain caution and adhere to validated susceptibility testing and regulatory guidelines until more definitive human data emerge.” We agree and similarly wrote in our discussion that “these results suggest that CAZ may remain an effective therapeutic option and that clinical testing is warranted to validate these pre-clinical findings” and “reiterate the need for clinical validation of *in vitro* susceptibility testing results, particularly if such results are going to be used to establish a standard of care for infections with limited therapeutic options.”
